# A Case Report of Iatrogenic Bronchial Rupture following Endobronchial Blocker Placement

**DOI:** 10.1155/2022/2494542

**Published:** 2022-05-23

**Authors:** Abdalhai Alshoubi, Asma Khan, Valerie DeJesus, Ellen Hauck

**Affiliations:** ^1^St. Joseph's Medical Center/ Dignity Health, 1800 N California St, Stockton, CA 95204, USA; ^2^Temple University Hospital, 3401 N Broad St, Philadelphia, PA 19140, USA

## Abstract

Physiologists Eduard Pfluger and Claude Bernard first introduced one lung ventilation (OLV) in 1871. Today, it is now a frequently used technique in open or minimally invasive cardiothoracic surgeries. One key benefit of the use of OLV is improved surgical exposure. Historically, lung isolation catheters used under fluoroscopic guidance or a Fogarty catheter were used to achieve OLV. In present times, endobronchial blockers (EBBs) in conjunction with single lumen endotracheal tubes and double lumen endotracheal tubes (DLTs) are used to achieve intraoperative OLV. Some complications of EBBs include mucosal injury, bleeding, bronchial rupture, pneumothorax, malpositioning-induced respiratory arrest, severe hypoxemia, and dislodgement. The incidence of iatrogenic tracheal rupture with single lumen endotracheal intubation is reported to be approximately 0.005%, and with double lumen ETT, the incidence may be between 0.05 and 0.19%. Mortality associated with tracheal rupture with DLTs is approximately 8.8%. Data on airway injury with endobronchial blockers is limited, and reported cases of bronchial perforations with use of EBBs are rare suggesting that EBBs may be the safer option for OLV. In this case report, we will be discussing a case of iatrogenic endobronchial rupture following endobronchial blocker placement.

## 1. Introduction

Physiologists Eduard Pfluger and Claude Bernard first introduced one lung ventilation (OLV) in 1871. Today, it is now a frequently used technique in open or minimally invasive cardiothoracic surgeries. One key benefit of the use of OLV is improved surgical exposure. Historically, lung isolation catheters used under fluoroscopic guidance or a Fogarty catheter were used to achieve OLV. In present times, endobronchial blockers (EBBs) in conjunction with single lumen endotracheal tubes and double lumen endotracheal tubes (DLTs) are used to achieve intraoperative OLV.

Lung separation devices are associated with minor complications, like sore throat and mucus membrane injury. We report a rare case of iatrogenic endobronchial rupture following endobronchial blocker placement.

## 2. Case Presentation

A 68-year-old pleasant woman presented with biopsy proven left lower lobe (LLL) adenocarcinoma stage IIIA pT1cN2M0 of the left lower lung. This patient had a longstanding history of asthma on Beta-2 agonist for bronchodilation, hyperlipidemia, and osteoporosis. She was a lifelong nonsmoker with no history of malignancy. Her weight was 78 kg with a height of 176 cm and a body mass index (BMI) of 25.2 kg/m. Positron emission tomography (PET) scan showed no metastasis. After thorough preoperative evaluation, the patient was scheduled for left bronchoscopy with left video-assisted thoracoscopy (VATS), left lower lobectomy, and mediastinal lymph node dissection.

The patient was intubated with a polyvinyl chloride 8.5 mm endotracheal tube (ETT) after induction with weight-based dosing of intravenous anesthetic agents. Anesthesia was maintained with sevoflurane and oxygen. Left lung isolation was achieved with 9.0 French ETT bronchial blocker, “uniblocker,” manufactured by Fuji Systems Corporation. There was no difficulty in placing the blocker under the guidance of fiberoptic vision. Uniblocker was placed inside the left mainstem bronchus, and it was confirmed by fiberoptic bronchoscopy. The uniblocker was secured in position with its inbuilt securing screw. The bronchial blocker cuff was inflated incrementally to 8 cc of air under the guidance of the fiberoptic vision; good lung isolation was achieved without the need to apply suction.

Surgery proceeded as planned. More than one hour into the surgery, the surgeon noticed protrusion of the tip and a small portion of balloon of the blocker through the left main stem bronchus into the surgical field. Intratracheal position of the EBB was reconfirmed by bronchoscopy, and EBB was found to be in good position in the bronchial lumen. The balloon was deflated, and the EBB was removed with no damage to the surrounding structures noted on inspection. Two-lung ventilation was resumed. Loss of tidal volume was noted on the ventilator, and the decision was made to surgically repair the defect in the bronchus. The bronchial wall defect was repaired with pericardial flap. The remainder of the planned surgery was completed. Reversal of neuromuscular blockade and extubation of the trachea was uneventful. The patient's postoperative course was uneventful, and she was discharged from the hospital in stable condition.

## 3. Discussion

OLV remains a challenge for anesthesiologists. Review of literature showed a similar case of a left mainstem bronchus perforation with the use of EBB [1] [2]. Intraoperative injury with bronchial blockers is a rare event. Incidence of traumatic injury with use of DLTs is more than with EBBs. The injury to trachea or main bronchus with DLTs could be related to difficult intubation, multiple attempts to adjust the position of a DLT, use of a stylet, oversized DLT, or cuff overdistention [3]. There are many risk factors that associated with bronchial perforation during OLV which include female gender, height <165 cm, operator inexperience, multiple attempts, old age, friable mucus membrane, chronic obstructive pulmonary disease, radiotherapy, and chronic steroid use [2] [4] [5].

In 1936, Magill achieved lung isolation by passing a tube with inflatable cuff at distal end alongside a single lumen ETT [6]. The use of a Fogarty catheter which consists of soft tip and low volume, high pressure balloon cuff, and Wiruthian blocker to achieve bronchial isolation is obsolete in current times [7]. Over the decades, designs of DLT and EBBs have been revised to improve their intraoperative use and decrease iatrogenic injury. Video DLT by VivaSight VDLT (ETView Medical Ltd., Misgav, Israel) is available and allows a continuous intraoperative visualization of the position of DLT during OLV via a camera attached over the tracheal lumen of the tube [8]. Several designs of BBs are available commercially for intraoperative lung isolation.

Both DLTs and EBBs are used for lung isolation intraoperatively during one lung ventilation. DLTs are preferred over EBBs because of ease of placement, ease of individual lung ventilation, isolation, and suctioning, and intraoperative position stability. Oo et al. published a systematic review and meta-analysis that discusses ease of use and faster placement of DLTs compared to EBBs. Another reason for preference of DLTs could be training experience of the anesthesiologist or institutional practice. Finally, EBBs are more expensive than DLT, a possible determinant of their wider use. However, the article also notes the association between large diameter DLTs and airway and vocal cord injuries. Variable clinical experience of anesthesiologists with limited use of DLT in difficult airways or rapid sequence induction (RSI) for lung isolation and need of exchange of DLTs with ETT for postoperative ventilation is suggestive that despite our one report of bronchial injury, EBBs may be the safer choice [1].

## 4. Conclusion

Complications from endobronchial blockers are rare and not extensively documented. This case highlights the necessity of anesthesia providers to be vigilant so that if complications during one lung ventilation with the use of endobronchial blockers should arise, they can be diagnosed and managed early and efficiently.
